# Molecular glues: Recent advances in cereblon substrate identification and mechanistic insights

**DOI:** 10.1002/smo2.70112

**Published:** 2026-09-21

**Authors:** Qingyun Guan, Xiaoyu Shi, Qian Yang, Xiangyi Jiang, Xinyong Liu, Peng Zhan

**Affiliations:** ^1^ Department of Medicinal Chemistry, State Key Laboratory of Discovery and Utilization of Functional Components in Traditional Chinese Medicine, Shandong Key Laboratory of Druggability Optimization and Evaluation for Lead Compounds, Shandong Basic Science Special Academic Zone (Pharmacy) School of Pharmaceutical Sciences Shandong University Jinan Shandong China; ^2^ State Key Laboratory of Natural and Biomimetic Drugs Peking University Beijing China; ^3^ State Key Laboratory of Druggability Evaluation and Systematic Translational Medicine (Tianjin Institute of Pharmaceutical Research) Tianjin China

**Keywords:** CRBN, mechanisms of action, molecular glues, protein‐protein interactions, substrate discovery

## Abstract

This highlight summarizes the recent advances in cereblon (CRBN) substrate discovery and mechanisms of action within the field of molecular glues. It emphasizes that molecular glue technology has transitioned from the stage of monofunctional targeted protein degradation to that of rational design and multidimensional regulation. Key recent breakthroughs include: (1) overcoming the limitations of canonical CRBN recognition motifs, identifying extensive novel substrates and unconventional recognition modes independent of fixed specific secondary structures; (2) reconceptualizing G protein‐coupled receptor (GPCR) signal transduction theory by demonstrating that molecular glues can stabilize GPCR megacomplexes to achieve sustained signal modulation, providing a novel strategy to overcome the challenge of traditional drug tolerance; (3) pioneering the concept of orthosteric molecular glue inhibitors, which expands the mechanistic modalities of molecular glues. Furthermore, this highlight delineates the therapeutic potential and mechanistic basis of molecular glues in antiviral therapies against HIV and the mpox virus, corroborating their core essence of inducing or stabilizing protein‐protein interactions. Finally, it summarizes current challenges facing the field, including target discovery, rational design, and druggability optimization, aiming to provide a theoretical reference for innovative research and development of molecular glues and their applications in chronic diseases and antiviral therapies.

## Graphical abstract

Molecular glues exert diverse core biological functions by inducing or stabilizing protein–protein interactions, extending far beyond the traditional scope of targeted protein degradation. Key breakthroughs include expanded cereblon substrate recognition, sustained G protein‐coupled receptor megacomplex signaling, and the first orthosteric molecular glue inhibitor targeting CSN5, opening novel therapeutic avenues for cancer, chronic diseases and antiviral therapy.

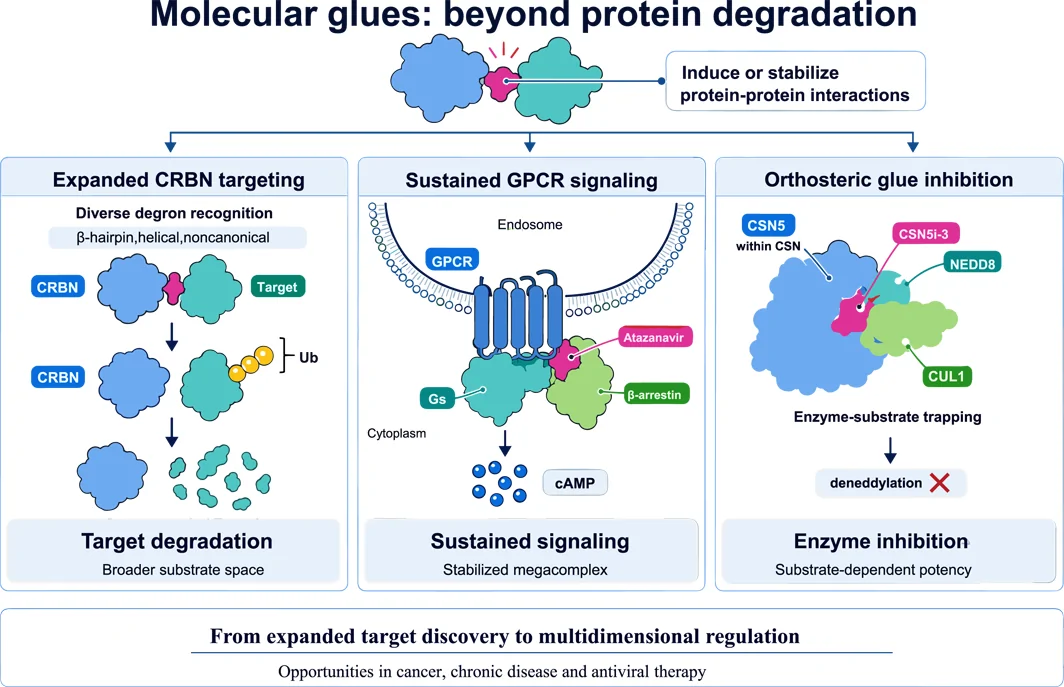

## 1 INTRODUCTION

**FIGURE 1:**
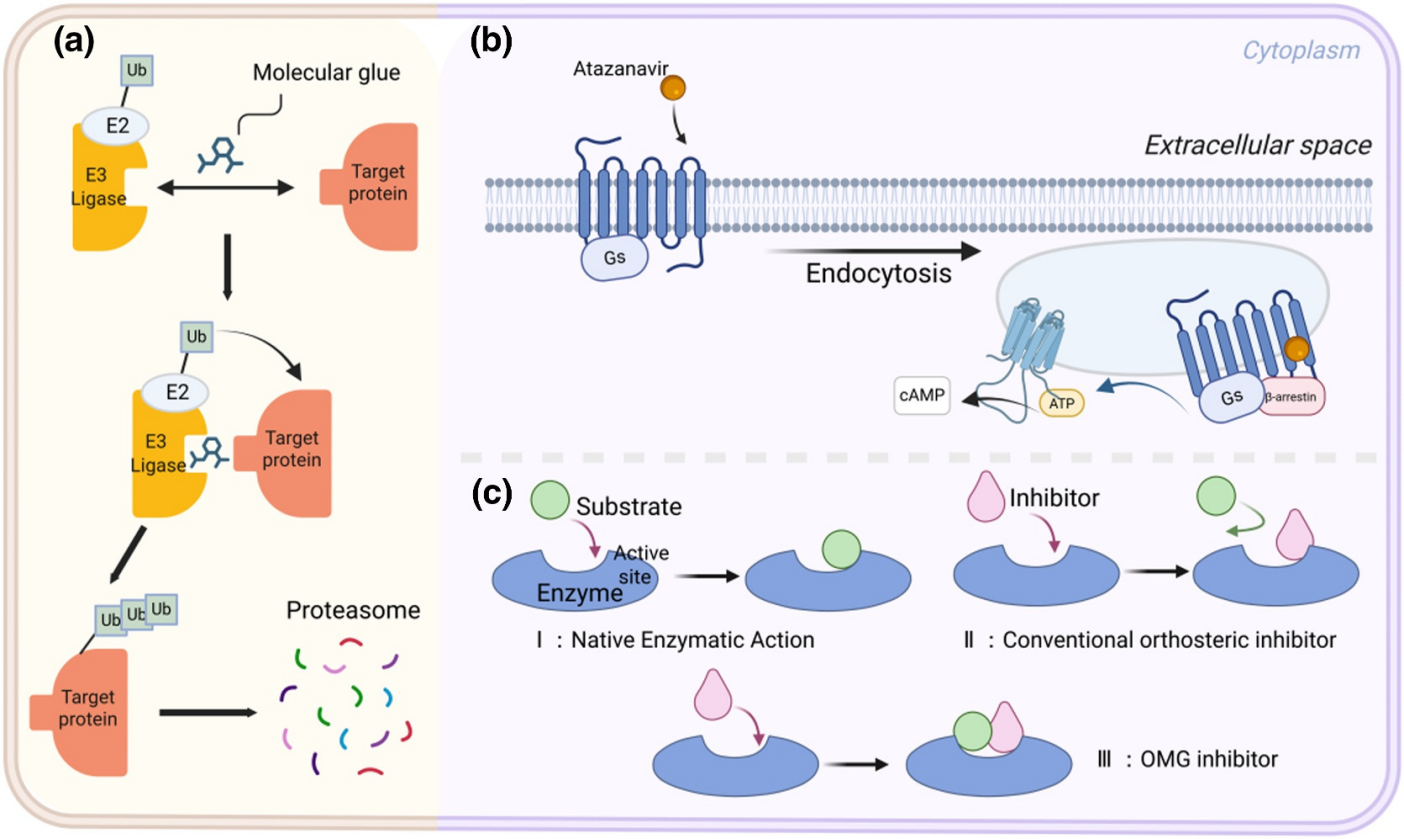
Diverse action modes of molecular glues. (a) Schematic of target protein degradation mediated by molecular glues; (b) atazanavir enables sustained GPCRs signaling by stabilizing the GPCR‐G protein‐*β*‐arrestin megacomplex; (c) comparison of three distinct modes of enzyme regulation, including native enzymatic action, conventional orthosteric inhibition, and OMG inhibition (Created with BioRender.com). GPCRs, G protein‐coupled receptors; OMG, orthosteric molecular glue.

Traditionally, molecular glues are a class of small‐molecule compounds that induce or stabilize protein‐protein interactions (PPIs) between a target protein and an E3 ubiquitin ligase, thereby mediating the ubiquitination and subsequent proteasomal degradation of the target protein (Figure 1a). In comparison with conventional enzyme inhibitors or receptor antagonists, molecular glues possess unique advantages, including the capacity to target undruggable proteins and modulate non‐enzymatic functions. Unlike proteolysis‐targeting chimeras (PROTACs), another major modality of targeted protein degradation (TPD) that rely on a dual‐ligand linker scaffold to bridge an E3 ligase and a target protein, molecular glues are monofunctional molecules without an artificial linker moiety. This structural feature endows molecular glues with generally lower molecular weight (typically <500 Da), more favorable membrane permeability, and superior oral bioavailability, while circumventing the synthetic complexity and pharmacokinetic limitations frequently associated with PROTAC linker design. Consequently, they are emerging as one of the most promising intervention strategies in the field of TPD therapeutics.[^1^, ^2^] The advent of thalidomide‐derived immunomodulatory drugs officially ushered in the era of systematic research and development of molecular glue degraders, with novel variants such as non‐degrading molecular glues gradually emerging in this process. Meanwhile, molecular glue technology has overcome the early bottlenecks of serendipity‐dependent discovery and monofunctional protein degradation restriction, and entered a new stage of rational design and multidimensional regulation.^[3]^

This article systematically summarizes the key breakthroughs in the field in recent years and conducts an in‐depth critical analysis. Specifically, it focuses on two core aspects: the systematic exploration of cereblon (CRBN) substrates and the mechanisms of action of molecular glues, providing an in‐depth dissection of how these relevant research achievements have expanded and revolutionized the field of molecular glues. Meanwhile, based on the long‐standing research foundation and academic interest of the authors' team in the field of antiviral drug research, this highlight further extends the discussion on the current application status, core mechanisms and developmental prospects of molecular glues in the field of antiviral drug research in the conclusion and perspectives, aiming to provide a more comprehensive theoretical reference and research inspiration for the rational design of next‐generation molecular glues and the innovative drug development in this direction.

## 2 SYSTEMATIC MINING OF CRBN SUBSTRATES AT THE PROTEOME‐WIDE LEVEL

Currently, as one of the most extensively studied E3 ubiquitin ligases in the field of molecular glues, CRBN exhibits marked limitations in the scope of its targetable proteins. Most of the reported canonical targets of CRBN molecular glues rely on the conserved recognition motif of the *β*‐hairpin G‐loop degron.^[4]^ This established perception has excluded a large number of proteins lacking the canonical *β*‐hairpin G‐loop motif from the targeting scope of existing CRBN molecular glues. Moreover, due to the long‐term lack of a systematic mining and prediction system for CRBN‐targetable substrates in the entire human proteome, the target discovery of molecular glues is highly dependent on phenotypic screening. As a result, a large number of undruggable targets closely associated with diseases remain consistently unable to be effectively intervened by molecular glues.

Petzold's team overcame these limitations through systematic proteome‐wide mining. Using the CK1*α β*‐hairpin G‐loop as a template, they performed structural matching across the Protein Data Bank and AlphaFold2 databases and predicted 1633 human proteins compatible with CRBN, including 1424 canonical *β*‐hairpin G‐loop proteins and 184 proteins bearing a newly identified helical G‐loop—a distinct CRBN recognition motif that engages CRBN via analogous hydrogen‐bonding interactions but uses a helical fold instead of *a β*‐hairpin. Beyond G‐loop‐dependent recognition, the team further modified the MaSIF‐seed algorithm from complementarity matching to surface similarity matching, enabling G‐loop‐independent substrate discovery. They confirmed that VAV1, a key autoimmune target lacking canonical G‐loops, binds CRBN by molecular surface mimicry of the GSPT1 degron. Ternary complex structures revealed that VAV1 uses the RT‐loop of its C‐terminal SH3c domain to form a hydrogen‐bond network with CRBN residues N351, H357, and W400, mimicking canonical G‐loop interactions without relying on defined secondary structures. These findings redefine the rules governing CRBN–substrate surface complementarity.^[5]^

In summary, this proteome‐wide substrate mining study not only provides tools for CRBN molecular glue target discovery, but also demonstrates that the *β*‐hairpin G‐loop motif is not essential for CRBN substrate recruitment, greatly expanding the targetable scope of molecular glues and laying a theoretical foundation for targeting undruggable proteins.

## 3 MOLECULAR GLUE‐MEDIATED GPCR SIGNAL REMODELING

The above research has expanded the scope of CRBN‐targetable proteins in the field of molecular glues, while the work described below has promoted the innovation and extension of the molecular glue drug concept. G protein‐coupled receptors (GPCRs) constitute the largest superfamily of cell‐surface membrane receptors in humans, and represent one of the most core targets of clinical drugs. Approximately 40% of FDA‐approved drugs act through modulating GPCRs,^[6]^ which are widely used in multiple therapeutic areas, including metabolic diseases, neurological disorders, and tumors. According to the canonical GPCR signaling theory, the G protein and *β*‐arrestin pathways function in a mutually exclusive manner. Upon agonist stimulation, the receptor initially binds to G protein via its intracellular core domain to mediate transient signaling. Subsequently, the receptor is phosphorylated by GPCR kinase, which recruits *β*‐arrestin to bind to the intracellular core domain of the receptor. *β*‐arrestin then competitively displaces G protein, terminates G protein‐mediated signaling, and simultaneously mediates receptor endocytosis and desensitization.^[7]^ This mode of action means that GPCR agonists frequently face critical clinical drawbacks, including receptor desensitization, drug tolerance, and short duration of pharmacological efficacy.

However, recent studies have found that certain class B GPCRs still exhibit sustained G protein signal transduction mediated by *β*‐arrestin after receptor endocytosis, which is incompatible with the canonical model. This led to the hypothesis that GPCRs may simultaneously engage G protein and *β*‐arrestin to form a GPCR–G protein–*β*‐arrestin megacomplex, yet its assembly mechanism and structural basis remain elusive. Nguyen et al. have now resolved the first high‐resolution cryo‐electron microscopy structure of the megacomplex, revealing a binding mechanism distinct from the canonical mode. In this architecture, *β*‐arrestin associates exclusively with the phosphorylated C‐terminal tail of the receptor in a “hanging” conformation, leaving the intracellular G protein–binding pocket unoccupied. Consequently, G protein can still bind productively and trigger downstream signaling. This finding provides the first molecular explanation for sustained endosomal G protein signaling by class B GPCRs, revises the traditional view that the binding of G protein and *β*‐arrestin to the receptor is mutually exclusive, and lays a critical structural foundation for GPCR signal regulation and advancing molecular glue drug development.^[8]^

Besides, He et al. introduced the molecular glue mechanism into class A GPCR signaling regulation for the first time, establishing a new paradigm for molecular glue drug development.^[9]^ Using a yeast survival pressure selection high‐throughput platform, they identified atazanavir—an FDA‐approved HIV protease inhibitor—as a pan‐positive allosteric modulator broadly active across class A GPCRs. Further mechanistic studies confirmed that atazanavir exhibits the typical functional characteristics of molecular glues. It binds to an intracellular allosteric pocket between transmembrane helix 6 (TM6) and transmembrane helix 7 (TM7) at the intracellular end of the receptor, rather than occupying the orthosteric site, and directly interacts with the 197‐loop of *β*‐arrestin1, thus acting as a molecular bridge between the receptor and *β*‐arrestin1. Structural analysis revealed that, mediated by atazanavir, *β*‐arrestin is recruited to the intracellular side of the receptor, and its finger loop region does not bind to the core pocket of the receptor, so that the binding of *β*‐arrestin no longer interferes with G protein coupling. This finding overturns the traditional view that G protein and *β*‐arrestin signaling are mutually exclusive in class A GPCRs. Instead, the molecular glue enables stable coexistence of both signals, driving sustained G protein signaling from endosomes even after receptor internalization (Figure 1b). Functionally, this mechanism successfully avoids receptor desensitization induced by traditional agonists, and provides a transformative strategy to overcome the long‐standing clinical challenge of drug tolerance faced by GPCR‐targeted drugs.

Overall, the above two studies complete the remodeling of the canonical framework of GPCR signal transduction at the conceptual level, providing a novel strategy for the development of next‐generation GPCR drugs. More importantly, they elucidate that the core essence of molecular glues is to induce and stabilize PPIs through small molecules to realize precise regulation of biological functions, rather than being restricted to the single dimension of protein degradation.

## 4 CSN5i‐3 REDEFINES OMG INHIBITORS

Shi et al. further expanded and elevated the concept of molecular glue in their latest research. As a key regulator of the ubiquitin‐proteasome system, the COP9 signalosome (CSN) specifically removes the neural precursor cell‐expressed developmentally downregulated protein 8 (NEDD8) modification from Cullin‐RING E3 ligases via its catalytic subunit CSN5, thereby maintaining cellular protein homeostasis.^[10]^ Notably, CRBN, the core E3 ligase substrate receptor discussed throughout this review, functions as a component of the CRL4 E3 ligase complex, whose activity and stability are directly modulated by CSN‐mediated deneddylation.^[11]^ CSN5i‐3, a well‐known CSN inhibitor, occupies the CSN5 catalytic active site as an orthosteric inhibitor, which binds preferentially to the enzyme–substrate complex over the free enzyme. Using structural, biochemical, and cellular approaches, this work definitively establishes CSN5i‐3 as the first bona fide orthosteric molecular glue (OMG) inhibitor (Figure 1c).^[12]^

High‐resolution ternary complex structures revealed that CSN5i‐3 does not merely act as a steric orthosteric blocker. Instead, it exerts a dedicated molecular‐glue function: it displaces the substrate isopeptide bond from the catalytic center, while its difluoromethylpyrazole moiety forms strong hydrophobic interactions with the C‐terminal tail of the excluded NEDD8, and its azepine moiety within the solvent‐exposed region contacts residue M721 in the winged‐helix B domain of Cullin1. This dual interfacial interaction drastically enhances the binding affinity between NEDD8 and CSN5. The NEDD8‐CSN interaction, undetectable without inhibitor, was markedly enhanced to approximately 130 nM by CSN5i‐3. Besides, the change in the C‐terminal tail of NEDD8 or the NEDD8‐binding moiety of CSN5i‐3 abolishes the glue effect and reduces the inhibitory potency of CSN5i‐3 to micromolar levels, validating the OMG mechanism.

Collectively, this study defines the structural and mechanistic basis of OMG inhibitors for the first time, verifying that enzyme orthosteric pockets can act as functional platforms for molecular glue action, and providing a novel conceptual framework for the development of substrate‐selective enzyme inhibitors. Moreover, this study is highly synergistic with molecular glue research in class A GPCRs. Together, the aforementioned study on GPCR signaling modulation and this study on OMG inhibitors overturn the restrictive view that molecular glues equal protein degraders, reaffirm the core nature of molecular glues as small‐molecule modulators of PPIs, and expand the conceptual and application scope of molecular glue technology.

## 5 CONCLUSION AND PERSPECTIVES

Collectively, the cutting‐edge advances reviewed herein have catalyzed a profound paradigm shift in the molecular glue field, unifying around a refined mechanistic core while dismantling long‐standing conceptual constraints. These breakthroughs converge on a pivotal redefinition: molecular glues are not confined to TPD but represent a versatile class of small molecules that precisely govern biological functions by inducing or stabilizing PPIs across diverse cellular and pathogenic contexts. From a functional evolution perspective, molecular glue technology has transcended its origins in monofunctional, ubiquitin‐dependent proteolysis. It now encompasses a multifaceted regulatory toolkit that reshapes GPCR signal transduction and drives substrate‐selective enzyme inhibition.

Beyond reshaping fundamental pharmacological principles, molecular glues have emerged as a transformative platform for antiviral innovation.^[13]^ In HIV‐1 therapy, allosteric integrase inhibitors act as molecular glues that induce the formation of an aberrant interface between the catalytic core domain and carboxy‐terminal domain of HIV‐1 integrase (IN). This abnormal integrase polymerization can inhibit viral maturation, thereby effectively suppressing virus replication, and has no antagonistic effect with clinically commonly used antiretroviral drugs. Compounds such as BDM‐2 and Pirmitegravir have completed phase I clinical trials and shown favorable safety and pharmacokinetic profiles.[^14^, ^15^] These HIV‐targeting agents represent one antiviral paradigm of molecular glues, which act by inducing non‐native, aberrant PPIs to disable viral protein function.

In contrast, the approved drug, tecovirimat, against mpox virus follows a distinct mechanism, exerting antiviral effects by stabilizing native viral protein oligomers. As a molecular glue, tecovirimat binds to the interfacial cavity of the homodimer of viral phospholipase F13, and stabilizes the F13 homodimer, blocking viral envelope formation and extracellular release. Drug‐resistant variants identified clinically, including A295E and 4MUT, confer resistance to tecovirimat by destabilizing the F13 dimer interface and abrogating drug‐induced dimerization, which underpin drug‐resistance monitoring and inform the design of novel antivirals.^[16]^ These studies in the antiviral field further validate the core essence of molecular glues as “inducing or stabilizing PPIs,” and define the key sites for molecular recognition through high‐resolution crystal structure determination (such as the 1.8–2.1 Å structure of the HIV‐1 IN‐ALLINI complex and the 2.6 Å structure of the F13‐tecovirimat complex), providing a paradigm for the rational design of agents targeting viral PPIs.

In the field of cancer therapy, molecular glues have emerged as a transformative therapeutic platform that enables the targeting of previously undruggable oncoproteins and the circumvention of acquired drug resistance. For B‐Raf proto‐oncogene, serine/threonine kinase (BRAF)‐mutant colorectal cancer, rationally designed CRBN‐based molecular glue degraders of HuR (dHuRs) recruit HuR by engaging its *β*‐hairpin G‐loop degron to trigger proteasomal degradation. Mechanistically, HuR depletion induces exon 18 skipping of BRAF pre‐mRNA, which downregulates oncogenic BRAF expression and durably suppresses mitogen‐activated protein kinase (MAPK) signaling. This mode of action exerts potent antitumor activity against both treatment‐naive and BRAF inhibitor‐resistant tumors. Furthermore, kinome‐wide clustered regularly interspaced short palindromic repeats, screening has validated that combining dHuRs with epidermal growth factor receptor, or MAPK kinase inhibitors markedly enhances cytotoxicity of dHuRs.^[17]^

In parallel, RAS(ON)‐targeting tri‐complex inhibitors (TCIs) represent another core molecular glue modality. These agents directly bind to GTP‐bound active rat sarcoma viral oncogene homolog (RAS) and recruit the endogenous chaperone protein cyclophilin A (CYPA) to assemble a stable RAS–inhibitor–CYPA ternary complex. This complex sterically occludes the effector‐binding interface of RAS, preventing the engagement of native downstream transducers such as rapidly accelerated fibrosarcoma kinase (RAF) and phosphatidylinositol 3‐kinase, and thus abrogating oncogenic RAS signaling. Investigations into clinical resistance to TCIs have deciphered two convergent mechanisms: Kirsten rat sarcoma viral oncogene homolog (KRAS) Y64 mutations directly disrupt π‐π stacking with inhibitors and impair ternary complex assembly, whereas KRAS Y71H or Class III (kinase‐dead or hypoactive) BRAF mutations indirectly confer resistance by reinforcing native RAS‐RAF interactions via neomorphic contacts and enhanced RAF dimerization. To surmount these resistance phenotypes, a next‐generation TCI, RMC‐4791, has been identified to overcome Y64‐driven resistance.^[18]^ These advances collectively underscore the core essence of molecular glues as PPI modulators, with high‐resolution structural determinations providing a critical blueprint for rational drug design.

Meanwhile, all the aforementioned studies have broken through the inherent mode of action in their corresponding fields. The research on CRBN substrate mining breaks through the restriction of canonical degron motifs on molecular glue targets; the GPCR‐related studies overturn the traditional theory of mutual exclusivity between G protein and *β*‐arrestin signaling pathways in Class A GPCRs; the study on CSN5 inhibitors proposes the concept of OMG inhibitors for the first time; and the antiviral molecular glue studies expand the application boundaries of molecular glues in the regulation of pathogen life cycles, proving that they can achieve precise intervention in the infection process by targeting virus‐specific PPI networks. Furthermore, these studies all take high‐resolution structural biology determination as the core support, elucidate the molecular mechanism of molecular glue action, lay a solid foundation for the transition of molecular glues from empirical screening to rational design, and also significantly broaden the range of targetable proteins for molecular glues, covering diverse target types, including intracellular proteins, viral enzymes, and membrane receptors.

Despite unprecedented progress, the field retains critical challenges that must be resolved to unlock full translational potential:

First, the target discovery and predictive modeling remain incomplete. Therefore, the proteome‐wide prediction and validation system for CRBN substrates should be improved. Based on the previously identified novel recognition motifs and surface complementarity rules, combined with protein structure prediction and AI algorithm optimization, a standardized workflow for target mining and precise molecular glue design should be established to reduce the reliance of target discovery on phenotypic screening. For antiviral molecular glues, it is necessary to further clarify the species specificity and conservation of viral PPI interfaces to provide a basis for the development of broad‐spectrum antiviral molecular glues.

Second, rational design frameworks for non‐degrading molecular glues are lacking. Universal design principles must be established for GPCR signal modulators, OMG inhibitors, and antiviral PPI stabilizers. Elucidating structure–activity relationships for aromatic stacking, hydrophobic clustering, and electrostatic interactions will guide optimization of affinity, selectivity, and functional efficacy. This will provide guidance for the targeted optimization of the binding affinity and selectivity of molecular glues, and further broaden the functional application boundaries of molecular glues.

Third, druggability and resistance bottlenecks persist. Aiming at the drug resistance issues faced by antiviral molecular glues (such as the T174I mutation of HIV‐1 IN and the ΔN267 deletion of F13), drug resilience can be improved through structure‐guided drug modification (e.g., replacing the tert‐butoxy group of BDM‐2 with a cyclopropoxy group to reduce drug resistance mediated by the T174I mutation). A pharmacodynamic and toxicological evaluation system tailored to the novel mechanisms of action should be established, with particular attention paid to the tissue distribution, barrier to viral drug resistance, and long‐term medication safety of antiviral molecular glues, so as to improve target selectivity and clinical translation potential.

The studies covered in this highlight, including CRBN substrate mining, GPCR signaling regulation, CSN5 orthosteric inhibition, and antiviral applications, have opened up entirely new avenues for the molecular glue field. This paradigm innovation in targeted therapy driven by molecular glues will not only provide new strategies for chronic diseases such as cancer and neurodegenerative diseases, but also open up precise intervention routes targeting drug‐resistant pathogens in the field of antiviral therapy, bringing brand‐new solutions for the treatment of major diseases.

## CONFLICT OF INTEREST STATEMENT

The authors declare no conflicts of interest.

## Data Availability

Data sharing not applicable to this article as no datasets were generated or analyzed during the current study.
